# Supplementary material for: Analysis of High Performance Concrete Mixed with Nano-Silica in Front of Sulfate Attack
Source: Materials (Basel). 2022 Oct 29;15(21):7614. doi: 10.3390/ma15217614 (PMC9659054; doi:10.3390/ma15217614)
Supplement: Supplementary file 1 [file materials-15-07614-s001.zip › materials-1904206-supplementary.pdf]

Table S1. The time-dependent mass loss of HPC specimens subject to sulfate erosion (means ± SD) (%)

Soaking time (d)	0% NS	1% NS	2% NS	3% NS	4% NS	5% NS
0	0	0	0	0	0	0
100	-0.175 ± 0.035	-0.0915±0.018	0.345±0.069	-0.467±0.094	-0.638±0.127	-0.791±0.128
200	0.723 ± 0.145	-0.0335±0.007	-0.292±0.058	-0.521±0.104	-0.805±0.161	0.975±0.162
300	3.494 ± 0.699	3.0230±0.605	3.689±0.738	3.948±0.789	4.153±0.831	4.421±0.831

Table S2. The time-dependent elastic modulus of HPC specimens subject to sulfate erosion from elastic wave test (means ± SD) (GPa)

Soaking time (d)	0% NS	1% NS	2% NS	3% NS	4% NS	5% NS
0	33.8± 0.676	35.7±0.714	33.4±0.668	33.7±0.674	34.5±0.690	32.9±0.658
100	31.7± 0.634	37.3±0.746	34.7±0.694	30.4±0.608	27.1±0.542	31.9±0.638
200	27.7 ± 0.554	36.3±0.726	30.1±0.602	27.4±0.548	26.1±0.522	28.5±0.570
300	23.2± 0.464	34.9±0.698	25.5±0.510	25.3±0.506	23.7±0.474	21.4±0.428

Table S3. The time-dependent elastic modulus of HPC specimens subject to sulfate erosion from mechanicals test (means ± SD) (GPa)

Soaking time (d)	0% NS	1% NS	2% NS	3% NS	4% NS	5% NS
0	29.3±0.585	31.9±0.638	29.2±0.583	29.7±0.595	30.2±0.605	28.3±0.566
100	27.8± 0.556	32.7±0.654	28.9±0.579	28.1±0.560	26.1±0.522	28.1±0.561
200	25.1 ± 0.502	30.5±0.610	27.1±0.542	26.8±0.537	23.7±0.474	23.2±0.463
300	21.8± 0.436	28.7±0.573	26.3±0.525	24.4±0.487	21.7±0.434	22.7±0.454

Table S4. The time-dependent compressive strength of HPC specimens subject to sulfate erosion (means ± SD) (MPa)

Soaking time (d)	0% NS	1% NS	2% NS	3% NS	4% NS	5% NS
0	68.4±1.368	71.9±1.438	73.1±1.463	65.8±1.316	62.5±1.250	62.5±1.214
100	60.2± 1.204	73.2±1.464	71.9±1.438	64.8±1.296	63.8±1.276	63.8±1.164
200	57.3± 1.146	66.8±1.336	53.7±1.074	48.5±0.970	42.1±0.840	42.0±1.096
300	30.2± 0.604	61.3±1.226	50.8±1.01	43.0±0.860	41.0±0.820	40.8±1.016

Table S5. The time-dependent splitting strength of HPC specimens subject to sulfate erosion (means ± SD) (MPa)

Soaking time (d)	0% NS	1% NS	2% NS	3% NS	4% NS	5% NS
0	5.9±0.118	6.5±0.13	6.9±0.138	5.5±0.11	5.2±0.104	5.3±0.106
100	6.6± 0.132	7.7±0.154	8.0±0.160	6.9±0.138	5.4±0.108	5.6±0.112
200	5.5± 0.110	7.4±0.148	6.0±0.120	6.2±0.124	5.3±0.106	5.1±0.102
300	4.3±0.086	7.2±0.144	5.4±0.108	5.3±0.105	4.7±0.094	4.6±0.092

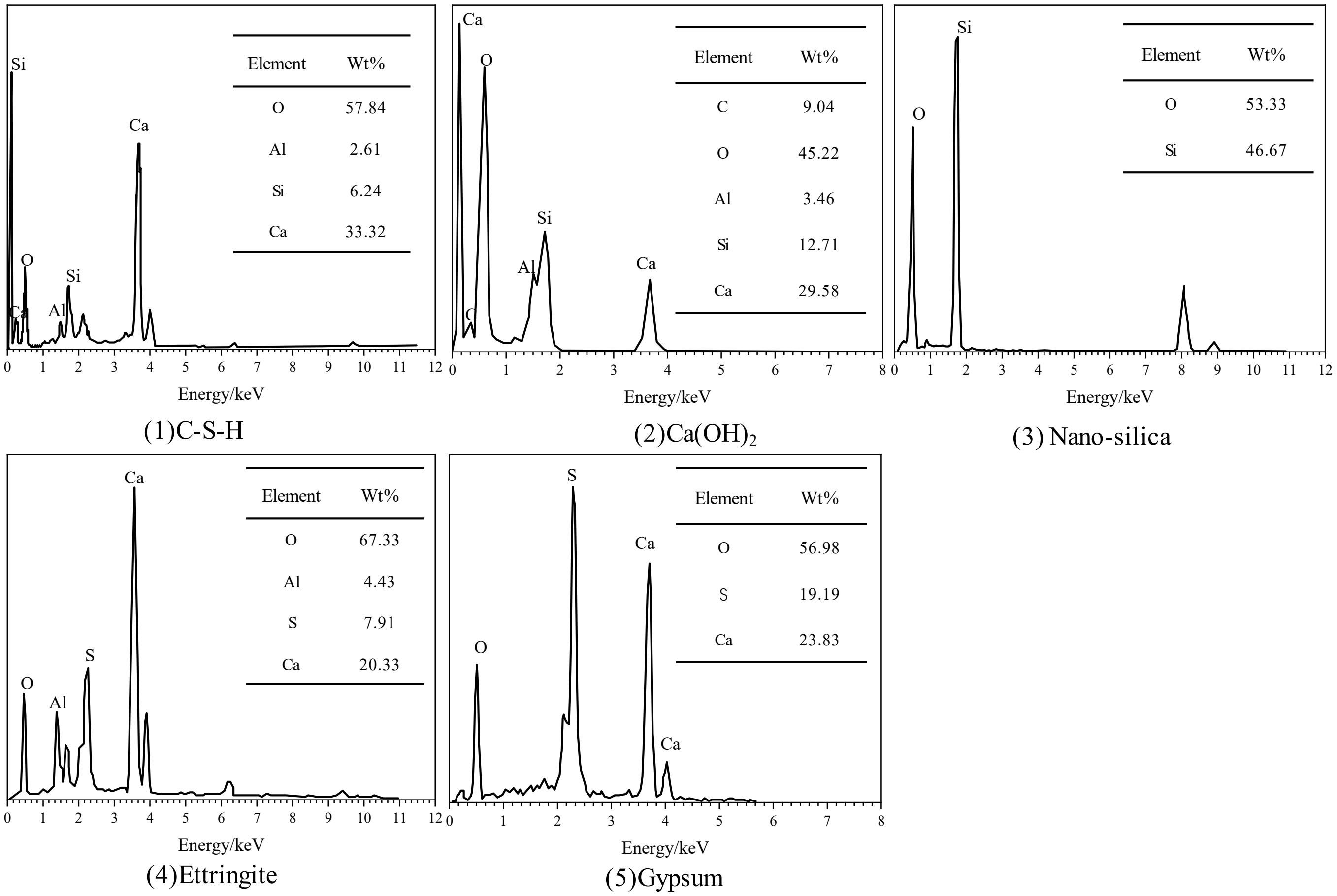

Figure S1. EDS results of different minerals
